# Colesevelam lowers glucose and lipid levels in type 2 diabetes: the clinical evidence

**DOI:** 10.1111/j.1463-1326.2009.01181.x

**Published:** 2010-05

**Authors:** Vivian A Fonseca, Yehuda Handelsman, Bart Staels

**Affiliations:** 1 Tulane University Health Sciences Center in New OrleansNew Orleans, LA, USA; 2 Metabolic Institute of AmericaTarzana, CA, USA; 3 Université Lille Nord de FranceLille, France; 4 INSERM U545Lille, France; 5 UDSLLille, France; 6 Institut Pasteur de LilleLille, France

**Keywords:** bile acid sequestrant, colesevelam HCl, dyslipidaemia, hyperglycaemia, low-density lipoprotein cholesterol, type 2 diabetes mellitus

## Abstract

Simultaneous control of blood glucose and other risk factors such as hypertension and dyslipidaemia is essential for reducing the risk of complications associated with type 2 diabetes mellitus (T2DM). As relatively few patients with T2DM have their risk factors managed to within the limits recommended by the American Diabetes Association, American College of Endocrinology or National Cholesterol Education Program Adult Treatment Panel III guidelines, treatment that can simultaneously control more than one risk factor is of therapeutic benefit. Clinical studies have shown that bile acid sequestrants have glucose-lowering effects in addition to their low-density lipoprotein cholesterol-lowering effects in patients with T2DM. The bile acid sequestrant colesevelam hydrochloride is approved as an adjunct to antidiabetes therapy for improving glycaemic control in adults with T2DM. This review examines data from three phase III clinical trials that evaluated the glucose- and lipid-lowering effects of colesevelam when added to the existing antidiabetes treatment regimen of patients with T2DM.

## Introduction

Heart disease and stroke are the leading cause of mortality in patients with diabetes, accounting for approximately 65% of deaths in this group [1,2]. The hyperglycaemia that accompanies uncontrolled type 2 diabetes mellitus (T2DM) is often associated with hypertension and dyslipidaemia, which combine to result in significant microvascular (retinopathy, nephropathy and neuropathy) and macrovascular [cardiovascular disease (CVD), stroke and peripheral arterial disease] complications that are the hallmark of T2DM [2–4]. Simultaneous control of hyperglycaemia, hypertension and dyslipidaemia is, therefore, essential for reducing the risk of complications in patients with T2DM.

Epidemiological evidence suggests that the presence of microvascular complications [predicted by glycosylated haemoglobin (HbA1c) levels] may increase the risk of developing macrovascular complications in patients with T2DM [5–7]. Although glycaemic control has been shown to have a clear role in reducing the risk of microvascular complications in patients with T2DM, the effect of glycaemic control on macrovascular disease risk is still under investigation [8,9]. Evidence from recent clinical trials suggests that intensive glucose-lowering therapy [treating to HbA1c target levels <6.0% (42.0 mmol/mol)] is not correlated with a reduction in CVD events [10,11]. Intervention to manage multiple risk factors (hyperglycaemia, hypertension and dyslipidaemia) concurrently in patients with T2DM can reduce the risk of CVD by approximately 50% [12]. However, as highlighted by data from the National Health and Nutrition Examination Survey evaluating trends between 1999–2000 and 2003–2004, there is still room for improvement in attaining simultaneous control of hyperglycaemia, hypertension and dyslipidaemia [13–15]. Specifically, while the proportion of patients achieving glycaemic target levels [HbA1c <7.0% (53 mmol/mol)], blood pressure (<130/80 mmHg), and total cholesterol [<200 mg/dl (<5.2 mmol/l)] increased from 35.8 to 57.1%, 35.7 to 48.3% and 48.8 to 50.4% respectively, the proportion of patients achieving all three treatment goals remained low, increasing from 7.5 to 13.2% [15]. These data indicate that there is still a clear need for intensive treatment to improve cardiovascular risk factor management in most patients with T2DM.

Bile acid sequestrants are well-established therapies for the treatment of hyperlipidaemia, and therapy with cholestyramine has been shown to contribute to reductions in both the progression of coronary heart disease and in the risk of CVD events [16,17]. Compared with the first-generation bile acid sequestrants (cholestyramine and colestipol), the second-generation bile acid sequestrant colesevelam hydrochloride (HCl) has a greater binding capacity for bile acids [18]. Clinical studies have shown that colesevelam monotherapy can lower low-density lipoprotein cholesterol (LDL-C) levels by 15–19% [19–21]. Furthermore, colesevelam can be safely combined with existing statin therapy in patients who would benefit from additional LDL-C lowering. Colesevelam, in combination with statin therapy, can result in LDL-C reductions of 42% (with simvastatin 10 mg) to 48% (with atorvastatin 10 mg) [22,23]. In 2000, the US Food and Drug Administration (FDA) approved the use of colesevelam for the treatment of hyperlipidaemia.

Clinical evidence has suggested that bile acid sequestrants could also improve glycaemic control in patients with T2DM [24–28], and this evidence was the basis for the approval of colesevelam by the US FDA in 2008 as an adjunct therapy for glycaemic control in adults with T2DM. This review examines the clinical data from these recent trials with colesevelam in patients with T2DM.

## Clinical Trials with Colesevelam HCl in Patients with T2DM

The addition of colesevelam to established antidiabetes monotherapy or combination therapy with metformin, sulfonylureas or insulin in patients with T2DM was evaluated in three randomized, double-blind, placebo-controlled clinical trials [26–28]. The trials enrolled a total of 1064 patients with T2DM with inadequate glycaemic control [HbA1c of 7.5–9.5% (58.5–80.3 mmol/mol), inclusive] on their current antidiabetes treatment regimen (table 1) [26–28]. Baseline glucose and lipid characteristics were similar among patients in the three trials, with baseline HbA1c levels ranging from 8.1 to 8.3% (65.0–67.2 mmol/mol) and baseline LDL-C levels ranging from 99.0 to 106.0 mg/dl (2.6 to 2.8 mmol/l) (table 1). The three populations were similar in demographics; mean age (approximately 56 years); male gender (51.0–56.0%); and race: Caucasian (56.0–64.0%), Black (10.0–19.0%) and Latino (16.0–29.0%). Compliance with study medication across the three trials ranged from 92.7 to 93.3% with colesevelam and 90.8 to 94.5% with placebo [26–28]. In addition, a similar proportion of patients were on statin therapy at baseline [137/316; 43.0% (metformin trial), 187/461; 40.6% (sulfonylurea trial), and 164/287; 57.1% (insulin trial)] [26–28].

**Table 1:** Trial design, patient baseline, and demographic criteria.

Colesevelam HCl or placebo added to	Metformin		Sulfonylurea		Insulin	
N	316		461		287	
Antidiabetes therapy	Metformin ± other oral antidiabetes agents		Sulfonylurea ± other oral antidiabetes agents		Insulin ± oral antidiabetes agents	
Design	Randomized, double-masked, placebo-controlled with 2-week single-blind placebo run-in		Randomized, double-masked, placebo-controlled with 2-week single-blind placebo run-in		Randomized, double-masked, placebo-controlled with 2-week single-blind placebo run-in	
Duration, weeks	26		26		16	
Patient baseline characteristics	Colesevelam HCl	Placebo	Colesevelam HCl	Placebo	Colesevelam HCl	Placebo
HbA1c, % (mmol/mol), mean ± s.d.	8.2 ± 0.7 (66.1 ± 15.8)	8.1 ± 0.6 (65.0 ± 16.9)	8.2 ± 0.7 (66.1 ± 15.8)	8.3 ± 0.7 (67.2 ± 15.8)	8.3 ± 0.6 (67.2 ± 16.9)	8.2 ± 0.6 (66.1 ± 16.9)
LDL-C, mg/dl (mmol/l), mean ± s.d.	106.0 ± 33.8 (2.7 ± 0.9)	99.0 ± 29.0 (2.7 ± 0.8)	104.3 ± 27.8 (2.7 ± 0.7)	106.0 ± 29.5 (2.7 ± 0.8)	102.0 ± 28.0 (2.6 ± 0.7)	102.0 ± 29.1 (2.6 ± 0.8)
Non-HDL-C, mg/dl (mmol/l), mean ± s.d.	189.9 ± 38.5 (4.9 ± 1.0)	181.0 ± 34.9 (4.7 ± 0.9)	142.5 ± 34.2 (3.7 ± 0.9)	142.7 ± 33.4 (3.7 ± 0.9)	135.7 ± 34.6 (3.5 ± 0.9)	139.0 ± 34.9 (3.6 ± 0.9)
Triglycerides, mg/dl (mmol/l), median ± IQR	172.3 ± 102.0 (1.9 ± 1.2)	166.0 ± 114.3 (1.9 ± 1.3)	177.0 ± 104.0 (2.0 ± 1.2)	173.3 ± 112.0 (2.0 ± 1.3)	155.0 ± 108.0 (1.8 ± 1.2)	167.0 ± 105.0 (1.9 ± 1.2)
Age, years, mean ± s.d.	56.0 ± 9.6	57.0 ± 9.5	57.0 ± 10.3	57.0 ± 10.3	57.0 ± 9.8	56.3 ± 9.3
Males, %	51.0	53.0	56.0	53.0	52.4	51.0
Caucasian, %	56.0	60.0	59.0	55.4	64.0	64.0
Black, %	15.0	17.0	10.0	15.0	16.3	19.0
Latino, %	25.0	20.0	29.0	26.0	17.0	16.0

HbA1c, glycosylated hemoglobin; HCl, hydrochloride; HDL-C, high-density lipoprotein cholesterol; IQR, interquartile range; LDL-C, low-density lipoprotein cholesterol; s.d., standard deviation.

### Trial 1: Colesevelam HCl Added to Metformin-Based Therapy [26]

#### Glucose Effects

**Figure 1:**
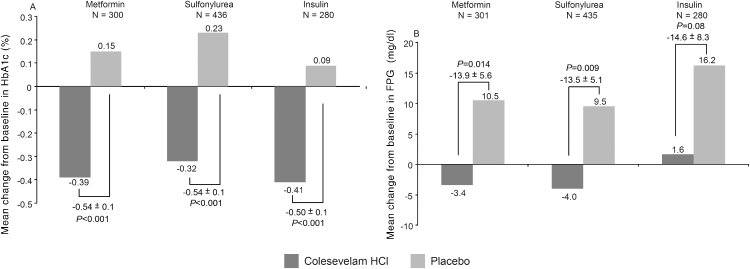
Mean change from baseline to endpoint LOCF in (A) HbA1c and (B) FPG with addition of colesevelam hydrochloride compared with placebo to ongoing metformin, sulfonylurea or insulin antidiabetes therapies in patients with T2DM. Endpoint was 26 weeks in the metformin and sulfonylurea trials, and 16 weeks in the insulin trial. Numbers above the bracketed pairs of bars are the mean treatment difference from baseline ± s.e. N is the number of patients with values at both baseline and endpoint. For HbA1c conversion from % to mmol/mol (%HbA1c—2.15*10.929); for FPG conversion from mg/dl to mmol/l, multiply by 0.0555. FPG, fasting plasma glucose; HbA1c, glycosylated haemoglobin; HCl, hydrochloride; LOCF, last observation carried forward; T2DM, type 2 diabetes mellitus.

When added to established metformin therapy, colesevelam resulted in further reductions in HbA1c at week 6 [−0.49 vs. −0.03% for placebo (−28.9 mmol/mol vs. −23.8 mmol/mol); p < 0.001]. By the end of the trial at week 26, the mean treatment difference (placebo-corrected change from baseline) in HbA1c was −0.54% (−29.4 mmol/mol; p < 0.001) (figure 1A). In addition, colesevelam was associated with a significant reduction in fasting plasma glucose at week 26 (treatment difference: −13.9 mg/dl [−0.8 mmol/l]; p = 0.014) (figure 1B). As a result, a significantly greater proportion of colesevelam-treated patients achieved the prespecified glycaemic goals [reduction in HbA1c ≥0.7% (≥15.8 mmol/mol) and/or fasting plasma glucose ≥30 mg/dl (1.7 mmol/l) at week 26] compared with placebo-treated patients (47.7 vs. 35.5%; p = 0.033).

A prespecified subgroup analysis investigated the effects of colesevelam when added to metformin monotherapy or metformin combination antidiabetes therapy. At week 26, the addition of colesevelam to metformin monotherapy resulted in a mean treatment difference in HbA1c of −0.47% (−28.6 mmol/mol; p = 0.002) while the addition of colesevelam to metformin in combination with other antidiabetes therapy resulted in a mean treatment difference in HbA1c of −0.62% (−30.3 mmol/mol; p < 0.001) (table 2). In this trial, the most common antidiabetes medications used in combination with metformin were sulfonylureas (69.4%) and thiazolidinediones (36.3%). These results suggest that colesevelam is versatile as an adjunctive treatment for T2DM, as it can be added early (to metformin monotherapy) or later in treatment (to antidiabetes combination therapy) to improve glycaemic control.

**Table 2:** Summary of glycosylated haemoglobin (HbA1c) reduction following addition of colesevelam hydrochloride (HCl) to ongoing diabetes monotherapy or combination therapy.

	Monotherapy			Combination therapy		
	Colesevelam HCl	Placebo	Treatment difference	Colesevelam HCl	Placebo	Treatment difference
Metformin trial, Bays et al. [26]						
N	79	76		69	76	
Change from baseline at week 26, %, (proportion)	−0.44 (−0.0044)	+0.02 (+0.0002)	−0.47^a^ (−0.0047)	−0.35 (−0.0035)	+0.27 (+0.0027)	−0.62^b^ (−0.0062)
Sulfonylurea trial, Fonseca et al. [27]						
N	69	80		149	138	
Change from baseline at week 26, %, (proportion)	−0.31 (−0.0031)	+0.48 (+0.0048)	−0.79^b^ (−0.0079)	−0.40 (−0.0040)	+0.02 (+0.0002)	−0.42^b^ (−0.0042)
Insulin trial^c^, Goldberg et al. [28]						
N	54	55		90	81	
Change from baseline at week 16, %, (proportion)	−0.43 (−0.0043)	+0.16 (+0.0016)	−0.59^b^ (−0.0059)	−0.41 (−0.0041)	+0.03 (+0.0003)	−0.44^b^ (−0.0044)

Combination antidiabetes therapy in the metformin trial was most commonly sulfonylureas (69.4%) and thiazolidinediones (36.3%); in the sulfonylurea trial, the most common medications were biguanides (68.8%) and thiazolidinediones (26.3%) and in the insulin trial, the most common medications used in combination were biguanides (60.8%) and thiazolidinediones (40.4%).

a p = 0.002.

b p < 0.001.

c Insulin only therapy or insulin therapy in combination with oral antidiabetes agents.

#### Lipid Effects

**Figure 2:**
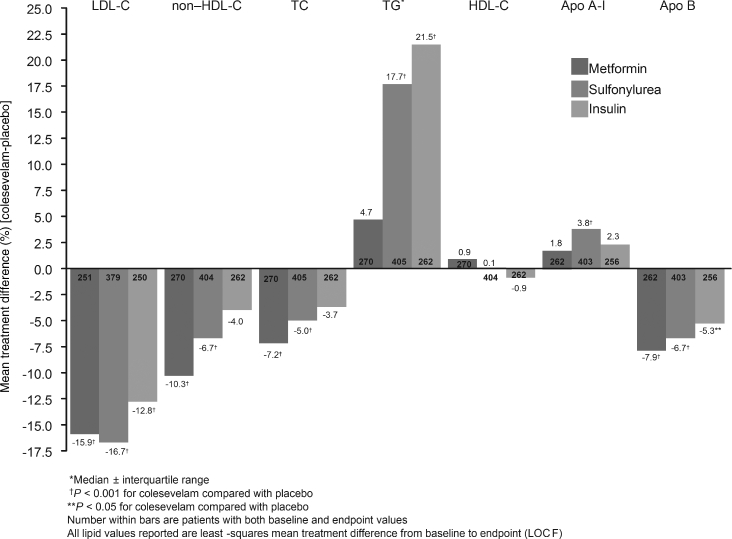
Mean change from baseline to endpoint in lipid parameters with addition of colesevelam HCl compared with placebo to ongoing metformin, sulfonylurea or insulin antidiabetes therapies in patients with T2DM. Mean values are reported unless otherwise indicated. Apo, apolipoprotein; HCl, hydrochloride; HDL-C, high-density lipoprotein cholesterol; LDL-C, low-density lipoprotein cholesterol; LOCF, last observation carried forward; LS, least-squares; TC, total cholesterol; T2DM, type 2 diabetes mellitus; TG, triglycerides.

The addition of colesevelam to metformin therapy resulted in a significant reduction in LDL-C of approximately 16% (p < 0.001) at week 26 (figure 2; table 3). In addition, there were significant reductions in total cholesterol and non-high-density lipoprotein cholesterol (non-HDL-C) following treatment with colesevelam compared with placebo (treatment difference: −7.2 and −10.3% respectively; p < 0.001 for both). The levels of HDL-C increased from baseline with both colesevelam and placebo by week 26, although the mean treatment difference between the groups was not significant (figure 2; table 3).

**Table 3:** Change from baseline in lipid and apolipoprotein levels and ratios following addition of colesevelam hydrochloride (HCl) or placebo to ongoing metformin, sulfonylurea or insulin therapy.

	Metformin—change at week 26		Sulfonylurea—change at week 26		Insulin—change at week 16	
	Colesevelam HCl	Placebo	Colesevelam HCl	Placebo	Colesevelam HCl	Placebo
LS mean percent change from baseline						
LDL-C	−12.3^a^	+3.7	−16.1^a^	+0.6	−12.3^a^	+0.5
TC	−4.1^a^	+3.1	−4.9^a^	+0.1	−3.1	+0.5
HDL-C	+1.1	+0.2	+0.5	+0.3	−0.5	+0.4
Non–HDL-C	−5.6^a^	+4.7	−6.1^a^	+0.6	−3.2	+0.8
TG^b^	+11.8	+6.6	+19.5^a^	+1.0	+22.7^a^	+0.3
Apolipoprotein A-I	+4.3	+2.5	+5.9^a^	+2.1	+4.7	+2.5
Apolipoprotein B	−4.0	+3.9	−5.9^a^	+0.8	−4.4^a^	+0.9
LS mean change in ratio						
TC/HDL-C	−0.21^a^	+0.17	−0.24^a^	0.00	−0.16	+0.01
LDL-C/HDL-C	−0.32^a^	+0.04	−0.44^a^	−0.01	−0.36^a^	−0.03
Non–HDL-C/HDL-C	−0.21^a^	+0.17	−0.24^a^	0.00	−0.16	+0.01
Apolipoprotein B/A-I	−0.07^a^	0.00	−0.10^a^	−0.01	−0.07^a^	−0.02

HDL-C, high-density lipoprotein cholesterol; LS, least-squares; LDL-C, low-density lipoprotein cholesterol; TC, total cholesterol; TG, triglycerides.

a p ≤ 0.004 vs. placebo.

b TG values reported as medians.

The effect of colesevelam on non-HDL-C levels is interesting given that non-HDL-C is considered a secondary target of lipid-lowering therapy in patients with T2DM with triglyceride levels ≥200 mg/dl (≥2.3 mmol/l) [29]. Non-HDL-C levels can provide a single, reliable index of atherogenic apolipoprotein (apo) B-containing lipoproteins, and guidelines from the third National Cholesterol Education Program Adult Treatment Panel recommend that non-HDL-C levels be lowered to <130 mg/dl (<3.4 mmol/l) to aid in the reduction of CVD events in patients with T2DM at highest cardiovascular risk, with an optional goal of <100 mg/dl (<2.6 mmol/l) in patients with high triglycerides [29–31].

There were improvements with colesevelam in the lipoprotein ratios that are most predictive of CVD risk [29,32]. These included reductions in the ratios of total cholesterol/HDL-C, LDL-C/HDL-C, non-HDL-C/HDL-C and apoB/apoA-I (p ≤ 0.002 vs. placebo for all). The clinical effect of these reductions was not examined in these trials.

**Figure 3:**
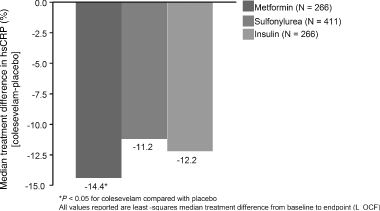
Change from baseline to endpoint in median high-sensitivity C-reactive protein (hsCRP) levels with addition of colesevelam HCl compared with placebo, to ongoing metformin, sulfonylurea or insulin antidiabetes therapies in patients with T2DM. N is the number of patients with values at both baseline and endpoint. HCl, hydrochloride; LOCF, last observation carried forward; T2DM, type 2 diabetes mellitus.

Patients treated with colesevelam exhibited a moderate, but non-significant increase in triglyceride levels compared with placebo (treatment difference: 4.7%; p = 0.221) (figure 2; table 3). According to the standards of care developed by the American Diabetes Association, patients with T2DM with unacceptable triglyceride levels [≥150 mg/dl (≥1.7 mmol/l)] may require additional therapy to manage their dyslipidaemia [8]. Interestingly, a similar proportion of patients in the colesevelam and placebo treatment groups who had a baseline triglyceride level <150 mg/dl (<1.7 mmol/l) had an increase in their triglyceride levels to ≥150 mg/dl (≥1.7 mmol/l) at any time during follow-up [12.1% (colesevelam group) vs. 12.5% (placebo group)]. In addition, 8.7% of patients in the colesevelam group and 5.9% of patients in the placebo group who had a baseline triglyceride level of ≥150 mg/dl (≥1.7 mmol/l) experienced a reduction in their triglyceride levels to <150 mg/dl (<1.7 mmol/l) after receiving study medication. Similar results were seen in the insulin and sulfonylurea trials with regard to triglyceride levels. Patients treated with colesevelam had a significant reduction in high-sensitivity C-reactive protein (hsCRP) levels compared with placebo (median treatment difference: −0.4 mg/l [−14.4%]; p = 0.02) at week 26 (figure 3) [26].

### Trial 2: Colesevelam HCl Added to Sulfonylurea-Based Therapy [27]

#### Glucose Effects

The addition of colesevelam to established sulfonylurea monotherapy or combination therapy resulted in a further reduction in HbA1c, with an overall mean treatment difference of −0.54% (−29.4 mmol/mol; p < 0.001) after 26 weeks (figure 1A). A significant reduction in HbA1c occurred when colesevelam was added to either sulfonylurea monotherapy (treatment difference: −0.79% [−32.1 mmol/mol]; p < 0.001) or sulfonylurea combination therapy [−0.42% (−28.1 mmol/mol); p < 0.001] (table 2). The addition of colesevelam to existing antidiabetes monotherapy or combination treatment suggests the potential benefit of its use in patients with early and established T2DM. Colesevelam also resulted in a significant mean treatment difference in fasting plasma glucose at week 26 (−13.5 mg/dl [−0.75 mmol/l]; p = 0.009) (figure 1B). The percentage of patients who achieved either a reduction in HbA1c ≥0.7% [≥15.8 mmol/mol] and/or fasting plasma glucose ≥30 mg/dl [1.7 mmol/l] at week 26 was greater in the colesevelam group compared with the placebo group (47.5 vs. 32.1% respectively; p = 0.001).

#### Lipid Effects

Colesevelam resulted in a significant mean treatment difference from baseline in LDL-C at week 26 (−16.7%; p < 0.001) (figure 2; table 3), as well as significant reductions in total cholesterol and non-HDL-C by the end of the trial (treatment difference: −5.0 and −6.7% respectively; p < 0.001 for both). While HDL-C levels increased from baseline with both colesevelam and placebo treatment, the changes were not significant. The total cholesterol/HDL-C, LDL-C/HDL-C, non-HDL-C/HDL-C and apoB/apoA-I ratios were significantly improved in the colesevelam group (p ≤ 0.003 vs. placebo). Compared with placebo, patients who received colesevelam had increased triglyceride levels [treatment difference: 17.7%; p < 0.001) (figure 2) and a non-significant reduction in hsCRP levels (median treatment difference: −0.4 mg/l (−11.2%); p = 0.06] from baseline to week 26 (figure 3) [27,33].

### Trial 3: Colesevelam HCl Added to Insulin-Based Therapy [28]

#### Glucose Effects

The addition of colesevelam to stable insulin only therapy or insulin therapy in combination with oral antidiabetes agents resulted in an additional reduction in HbA1c [treatment difference: −0.50% (−29.0 mmol/mol); p < 0.001] (figure 1A) after 16 weeks. This reduction in HbA1c with colesevelam was apparent and significant from the first assessment at week 4 [−0.32 vs. −0.02% for placebo (−27.0 vs. −23.7 mmol/mol); p < 0.001]. In addition, the reduction in HbA1c with colesevelam was significant whether colesevelam was added to ongoing insulin only therapy [−0.59% (−29.9 mmol/mol); p < 0.001] or insulin therapy in combination with oral antidiabetes agent(s) [−0.44% (−28.3 mmol/mol); p < 0.001] (table 2). Although fasting plasma glucose was reduced from baseline with colesevelam, the mean treatment difference was not significant at week 16 [−14.6 mg/dl (−0.8 mmol/l); p = 0.08] (figure 1B). Overall, a greater proportion of patients in the colesevelam group compared with the placebo group achieved either a reduction in HbA1c ≥0.7% (≥15.8 mmol/mol) and/or fasting plasma glucose ≥30 mg/dl (1.7 mmol/l) at week 16 (48.6 vs. 31.6% respectively; p = 0.004).

#### Lipid Effects

At week 16, colesevelam significantly reduced LDL-C levels as evidenced by a mean treatment difference of −12.8% (p < 0.001). Although the addition of colesevelam to insulin therapy also resulted in reductions from baseline in total cholesterol, non-HDL-C, and HDL-C at week 16 (figure 2; table 3), these reductions were not significant compared with placebo. While improvement was seen in the lipoprotein ratios conferring greatest cardiovascular risk in the colesevelam group, the difference between groups was significant only for the ratios of LDL-C/HDL-C (p < 0.001) and apoB/apoA-I (p = 0.004). In addition, patients treated with colesevelam exhibited a significant increase in triglyceride levels compared with placebo (treatment difference: 21.5%; p < 0.001) (figure 2; table 3). There was a non-significant reduction in hsCRP levels at week 16 last observation carried forward with colesevelam compared with placebo [median treatment difference: −0.4 mg/l (−12.2%); p > 0.05] (figure 3), however, the reduction in hsCRP levels was significant in the cohort of patients who completed the study at week 16 [median treatment difference: −0.6 mg/l (−18.6%); p ≤ 0.01] [28,33].

## Colesevelam HCl Provides Dual Lipid- and Glucose-Lowering Benefits in Patients with T2DM

The addition of colesevelam resulted in a consistent reduction in HbA1c [ranging from 0.50–0.54% (−29.0 to −29.4 mmol/mol)] across all three trials, regardless of the background antidiabetes medication used, that was accompanied by reductions in fasting plasma glucose. Furthermore, colesevelam treatment resulted in a greater percentage of patients achieving either a reduction in HbA1c ≥0.7% (≥15.8 mmol/mol) and/or fasting plasma glucose ≥30 mg/dl (1.7 mmol/l) at study endpoint compared with placebo. Colesevelam also reduced LDL-C and non-HDL-C levels when added to ongoing antidiabetes therapies, regardless of whether subjects were on ongoing monotherapy or combination therapy regimens. Colesevelam increased triglyceride levels; however, these increases were accompanied by reduced LDL-C, total cholesterol, and non-HDL-C levels, and increased HDL-C and in the lipoprotein ratios that typically denote increased cardiovascular risk. These results highlight the dual ability of colesevelam to improve both HbA1c and LDL-C in patients with T2DM.

## Safety of Colesevelam HCl in Combination with Existing Antidiabetes Therapy

Safety is an important issue in patients with T2DM who are often taking multiple medications to address the overall pathology of insulin resistance. Colesevelam, unlike most new pharmacological agents, has an established safety record and was generally well tolerated across all three trials in patients with T2DM. The rate of adverse events (AEs) was similar between the colesevelam and placebo groups, and most AEs were considered unrelated to the study medication (table 4). Gastrointestinal disorders were the most common drug-related AEs experienced with colesevelam and included constipation (occurring in 6.1–7.0% of patients) and dyspepsia (occurring in 2.2–3.4% of patients) [26–28]. In the metformin trial, six patients (3.8%) and two patients (1.3%) receiving colesevelam and placebo respectively, withdrew because of drug-related AEs. In the sulfonylurea trial, 12 patients (5.2%) and four patients (1.7%) receiving colesevelam and placebo respectively, and five patients (3.4%) and two patients (1.4%) in the insulin trial receiving colesevelam and placebo respectively, withdrew because of drug-related AEs [26–28]. Most AEs were mild-to-moderate in severity and none of the serious AEs were considered to be drug-related.

**Table 4:** The incidence of adverse events (AEs) following addition of colesevelam hydrochloride (HCl) or placebo to ongoing metformin, sulfonylurea or insulin therapy.

	Metformin		Sulfonylurea		Insulin	
	Colesevelam HCl (N = 159)	Placebo (N = 157)	Colesevelam HCl (N = 229)	Placebo (N = 231)	Colesevelam HCl (N = 147)	Placebo (N = 140)
All AEs, N (%)	85 (54.0)	81 (52.0)	145 (63.3)	126 (55.0)	92 (63.0)	82 (59.0)
Drug-related AEs, N (%)	29 (18.2)	14 (9.0)	47 (21.0)	21 (9.1)	24 (16.3)	13 (9.3)
Serious AEs, N (%)	8 (5.0)^a^	5 (3.2)^a^	8 (4.0)^a^	11 (5.0)^a^	11 (8.0)^a^	8 (6.0)^a^
Most common drug-related AEs (occurring in ≥2% of patients)						
Constipation, n (%)	11 (7.0)	2 (1.3)	14 (6.1)	6 (3.0)	10 (6.8)	0
Dyspepsia, n (%)	5 (3.1)	5 (3.2)	5 (2.2)	1 (0.4)	5 (3.4)	0
Diarrhoea, n (%)	2 (1.3)	4 (3.0)	3 (1.3)	1 (0.4)	NA	NA
Flatulence, n (%)	2 (1.3)	1 (1.0)	2 (1.0)	3 (1.3)	3 (2.0)	0
Hypoglycaemia, n (%)	1 (1.0)	0	4 (2.0)	2 (1.0)	5 (3.4)	8 (6.0)

AEs and serious AEs occurring during the randomized phase of each trial. The randomized period was 16 weeks in the insulin trial and 26 weeks in both the metformin and sulfonylurea trials. AEs, adverse events; NA, not applicable.

a Not drug-related.

The risk of hypoglycaemia is an important consideration for any antidiabetes agent. In these three trials, colesevelam did not significantly increase the risk of hypoglycaemia when added to existing metformin-, sulfonylurea- or insulin-based therapy in patients with T2DM. In the metformin trial, one patient who received colesevelam experienced a mild episode of hypoglycaemia [26]. In the sulfonylurea trial, four patients (2.0%) in the colesevelam group and two patients (1.0%) in the placebo group developed hypoglycaemia that was considered drug-related [27]. In the insulin trial, five patients (3.4%) treated with colesevelam and eight patients (6.0%) treated with placebo reported hypoglycaemia that was considered to be drug-related [28]. Overall, most episodes of hypoglycaemia were mild and resolved without discontinuation of treatment.

Mean changes in safety laboratory parameters and vital signs were similar in the treatment groups of each trial [26–28]. Overall, colesevelam was found to be weight neutral when added to existing antidiabetes treatment, which is another important consideration for patients with T2DM.

Colesevelam can increase triglyceride levels in patients with T2DM. Caution is therefore recommended in patients with triglyceride levels >300 mg/dl (>3.4 mmol/l), and colesevelam is contraindicated in patients with triglyceride levels >500 mg/dl (>5.7 mmol/l) and in patients with a history of hypertriglyceridaemia-induced pancreatitis [21]. Colesevelam has a high capacity for bile acid binding with a low potential for interfering with the absorption of other agents [34]. However, patients taking levothyroxine, oral contraceptives or glyburide should take these agents at least 4 h before colesevelam to avoid any potential for impaired absorption. Use of colesevelam may also decrease the absorption of fat-soluble vitamins including A, D and E [21].

The results of these trials are similar to those seen following the addition of thiazolidinediones to metformin or sulfonylurea therapy [35]. Factors that may have influenced the efficacy of colesevelam in these trials (when compared with other agents) include the low mean baseline HbA1c and the fact that there was no ‘washout’ in the colesevelam studies [36]. Currently, there are no data to show whether use of colesevelam reduces mortality or morbidity in patients with T2DM. Colesevelam is not approved for use in patients with type 1 diabetes and has not been studied in combination with the currently approved dipeptidyl peptidase-IV inhibitors sitagliptin or saxagliptin. In addition, there are limited data on its use in patients receiving thiazolidinediones.

## Bile Acid Sequestrants: Mechanism(s) of Action for Their Glycemic Effects

The mechanism(s) by which colesevelam lowers glucose levels in patients with T2DM is not yet clearly understood. However, there is increasing evidence that the glycaemic effects of bile acid sequestrants may occur through farnesoid X receptor (FXR/bile acid receptor), liver X receptor, fibroblast growth factor-19 and TGR5-mediated effects on intestinal glucose absorption and/or hepatic glucose metabolism, in addition to influences on peripheral insulin sensitivity, incretin effects and energy homeostasis. Bile acid activation of FXR has been shown to reduce expression of genes involved in gluconeogenesis including phosphoenolpyruvate carboxykinase and glucose-6-phosphatase. In addition, FXR may modulate hepatic glucose production during fasting and postprandial hepatic glucose utilization [37–40]. Alterations in the bile acid pool in T2DM and its effect(s) on FXR activation are still under investigation. Emerging data suggest a partial regulatory role for FXR modulators in peripheral insulin sensitivity, suggesting a future role for FXR in the treatment of insulin resistance and T2DM [41–43]. Bile acids may also affect incretin release, having been shown to induce secretion of glucagon-like peptide-1 (GLP-1) through activation of the G-protein-coupled receptor TGR5 [44,45]. The bile acid sequestrant colestimide was shown to result in increased secretion of GLP-1 in patients with T2DM, although the functional consequences are unclear [46]. Bile acids have also been implicated in metabolic regulation, through FXR-mediated regulation of energy substrate mobilization and storage [47]. These glycaemic effects appear to be unique to bile acid sequestrants, within which only colesevelam has been approved for improving glycaemic control in adults with T2DM. These effects have not been observed with the cholesterol absorption inhibitor ezetimibe. Despite these advances, further study is needed to determine the precise mechanism underlying the effect of bile acid sequestrants on glucose metabolism in patients with T2DM.

## Conclusion

The additional reduction in HbA1c and LDL-C levels achieved with the addition of colesevelam to current antidiabetes therapies may help patients with T2DM achieve target levels for HbA1c and LDL-C. Favourable modification of these two factors plus lowering non-HDL-C levels may make colesevelam a useful adjunctive therapy to reduce overall cardiovascular risk in patients with T2DM. Colesevelam is currently recommended for the treatment of T2DM in combination with antidiabetes therapies such as metformin, sulfonylurea and insulin, and has been added to the treatment roadmap developed by the American Association of Clinical Endocrinologists [48], and to the clinical guideline for pharmacological management of T2DM developed by the Joslin Diabetes Center and Joslin Clinic [49].
